# Construction of lncRNA and mRNA co-expression network associated with nasopharyngeal carcinoma progression

**DOI:** 10.3389/fonc.2022.965088

**Published:** 2022-07-26

**Authors:** Xu Lu, Xing Chen, Xinke Wang, Jing Qing, Ji Li, Yunyun Pan

**Affiliations:** ^1^ Ningbo First Hospital, Ningbo, China; ^2^ Ninghai County Third Hospital, Ningbo, China

**Keywords:** lncRNA, nasopharyngeal carcinoma, SMAD5-AS1, co-expression network, WGCNA

## Abstract

Nasopharyngeal carcinoma is a type of head and neck cancer with a high incidence in men. In the past decades, the survival rate of NPC has remained around 70%, but it often leads to treatment failure due to its distant metastasis or recurrence. The lncRNA-mRNA regulatory network has not been fully elucidated. We downloaded the NPC-related gene expression datasets GSE53819 and GSE12452 from the Gene Expression Omnibus database; GSE53819 included 18 NPC tissues and 18 normal tissues, and GSE12452 included 31 NPC tissues and 10 normal tissues. Weighted gene co-expression network analysis was performed on mRNA and lncRNA to screen out modules that were highly correlated with tumor progression. The two datasets were subjected to differential analysis after removing batch effects, and then Venn diagrams were used to screen for overlapping genes in the module genes and differential genes. The lncRNA-mRNA co-expression network was then constructed, and key mRNAs were identified by MCODE analysis and expression analysis. GSEA analysis and qRT-PCR were performed on key mRNAs. Through a series of analyses, we speculated that BTK, CD72, PTPN6, and VAV1 may be independent predictors of the prognosis of NPC patients.Taken together, our study provides potential candidate biomarkers for NPC diagnosis, prognosis, or precise treatment.

## Introduction

Nasopharyngeal carcinoma is a type of head and neck cancer (1). NPC is highly metastatic, often metastasizing to local and distant lymph nodes, bone, lung, and liver (2, 3). NPC is relatively rare in North America and Europe but is commonly found in many places (4). NPC has significant gender differences, with men more likely to develop NPC (5). In addition to genetic susceptibility and environmental factors, NPC is caused by genetic and epigenetic alterations, as well as dense lymphatic infiltration of the primary tumor (6). With advances in radiation therapy techniques in recent decades, NPC has maintained a survival rate of about 70% with radiation therapy-based combination therapy, but tumor recurrence or distant metastases occur within a few years after treatment, leading to treatment failure (7). It is necessary to study the underlying molecular mechanisms of NPC occurrence to provide better biomarkers for the diagnosis and treatment of NPC. Recent researched have identified a variety of lncRNAs that are closely associated with various processes of tumorigenesis and progression (8, 9).

LncRNAs are long-stranded noncoding RNAs that play key roles in various cellular and physiological processes and are aberrantly expressed in various cancers (10). MEG3 overexpression promotes BCa cell apoptosis and inhibits cell proliferation (11). lncRNA ANRIL and lncRNA n375709 have also been shown to play a role in NPC by Zou et al. and Ren et al. (12, 13). However, our understanding of cancer-related lncRNAs is still very limited. One of the basic molecular mechanisms of lncRNAs is as competing endogenous RNAs, which are mutually regulated with microRNAs (miRNAs) and jointly participate in the expression of target gene mRNAs, playing this important role in tumor development. Therefore, cancer-critical functional lncRNAs can be identified by lncRNA-induced transcriptional disruption of target gene mRNAs (14, 15). The lncRNA-mRNA regulatory network related to NPC progression has been rarely reported due to the lack of common analysis of lncRNA and mRNA expression levels in NPC.

In this study, based on the NPC-related gene expression datasets GSE53819 and GSE12452, we performed weighted gene co-expression network analysis and differential analysis on the samples from them. The lncRNA-mRNA co-expression network related to NPC progression was constructed to explain the roles of NPC-associated mRNAs and lncRNAs. This finding gives potential biomarkers for NPC diagnosis, prognosis, or precise treatment.

## Methodology

### Data sources

We used the keyword “nasopharyngeal carcinoma” to find relevant datasets in the GEO database, and then manually reviewed and selected cohorts containing lncRNA and mRNA expression and clinical survival information, including 18 NPC tissues and 18 normal tissues, and the GSE12452 dataset (platform GPL570, Human mRNA) from the University of Wisconsin-Madison, including 31 NPC tissues and 10 normal tissues. Both datasets contain lncRNA and mRNA genes.

### WGCNA

Pearson correlation coefficients between genes were obtained from the differentially expressed mRNAs and lncRNAs used in WGCNA using the WGCNA database of R software; subsequently, an appropriate soft threshold β was chosen to ensure that the network was not scalable. A gene network is built to transform the adjacency matrix into a topological overlap matrix to generate a hierarchical clustering tree of genes. Highly correlated co-expressed gene modules were identified using the DynamicTreeCut method with thresholds set to cut height = 0.25 and minSize = 150. Pearson correlation test can analyze the relationship between module eigen genes and clinical features.

### Differential expression analysis

The GSE53819 dataset and GSE12452 dataset were subjected to batch effect removal using the R package, and the final matrix with batch effects removed was obtained. The differential expression of gene in this matrix was then conducted using the limma package of R software. “adj. P<0.05 and FC=0.07” were defined as the thresholds for lncRNA and mRNA differential expression screening, and heat maps and volcano maps were plotted using the pheatmap and ggplot2 packages, respectively.

### Venn analysis

The modules and differential genes screened by WGCNA were screened for overlapping genes using online calculations and plotting custom Venn diagrams.

### Protein-protein interaction network construction

Protein-protein interaction network construction was performed using the Metascape tool, and the MCODE algorithm was used to cluster the PPI network, identify potential protein complexes, and screen for hub genes in the PPI network. The mRNAs in the largest sub-networks were screened as key mRNAs. lncRNA and hub mRNA co-expression networks were constructed in Cytoscape software.

### GSEA

To find the effect of gene expression on NPC, we divided the samples from the combined expression profile into high and low expression parts and used GSEA analysis to obtain the key gene-related pathways. P-value < 0.05, FDR < 0.25, NES > 1 or less than -1 were used as screening conditions.

### Cell culture

The human nasopharyngeal carcinoma cell line SUNE-1 and human nasopharyngeal carcinoma epithelial cells NP69 were purchased from Cell Bank of Type Culture Collection supplemented with 10% fetal bovine serum, 100 U/mL penicillin, and 100 μ g/mL streptomycin. NP69 cells were cultured in a keratinocyte/serum-free medium. All cell lines were cultured in a humidified incubator at 37°C, 5% CO_2_.

### qRT-PCR

Total RNA was extracted from NP69 and SUNE-1 cells using TRIzol reagent, and lncRNA and mRNA were reverse transcribed to cDNA using FastQuant RT kit including gDNase and SuperReal Premix Plus-SYBR Green. Green to reverse transcribe lncRNA and mRNA to cDNA. real-time qPCR assays were performed using the miScript SYBR Green PCR Kit. The relative expression of the genes was calculated by the 2 -ΔΔCT method. The experiment was repeated three times and the mean was taken. gAPDH was used as an endogenous control for mRNA normalization. The primers are displayed in **Table 1**.

**Table 1:** qRT-PCR primers.

Genes	Primer sequences (5’- 3’)
BTK	F:CCAATGGCTGCCTCCTGAACTAC R:TCGGTGAAGGAACTGCTTTGACTC
CD72	F:CATCTCCAGCAGGTTAGGACA R:CGGGCACTTGAACATTCTC
PTPN6	F:TGCAGGGACGTGACAGTAAC R:TGACACGAGTGTTCTCCTGC
VAV1	F:TCAGTGCGTGAACGAGGTCAAG R:CCATAGTGAGCCAGAGACTGGT
GAPDH	F:GTCAACGGATTTGGTCTGTATT R: AGTCTTCTGGGTGGCAGTGAT

## Results

### Gene co-expression modules

**Figure 1:**
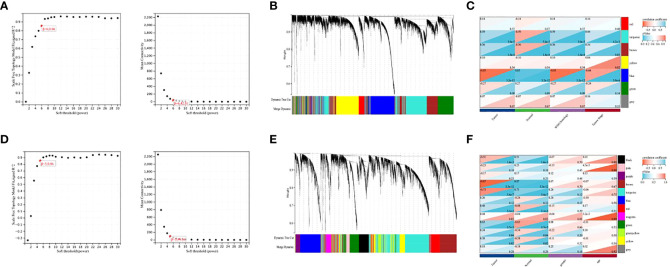
Identification of gene co-expression modules. **(A, D)** Network topologies with different soft threshold power; **(B, E)** Gene tree graph obtained based on overlapping clustering of the same topologies; **(C, F)** Correlation of each module with clinical information.

To explore the co-expression patterns of mRNA and lncRNA in NPC, we performed WGCNA analysis on the GSE12452 dataset and GSE53819 dataset, respectively. To ensure scale-free networks, we chose soft thresholds of β = 6, and β = 5, respectively (**Figures 1A, D**), used WGCNA packages as soft threshold power to generate hierarchical clustering trees (**Figures 1B, E**), and then we built co-expression networks of associations between clinical features and these modules (**Figures 1C, F**). The blue module and the turquoise module of GSE12452 were significantly associated with tumor progression, and these two modules were defined as SUR1 modules. the brown module and the turquoise module of GSE12452 were significantly associated with tumor progression, and these two modules were defined as SUR2 modules.

### Differential expression analysis

**Figure 2:**
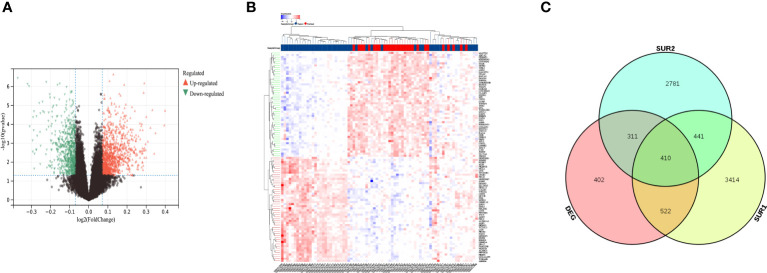
Identification of differentially expressed mRNAs and lncRNAs. **(A)** Volcano map showing differentially expressed lncRNA and mRNA; **(B)** Heat map showing differentially expressed lncRNA and mRNA; **(C)** Venn diagram screening SUR1, SUR2 module genes and overlapping genes of differential genes.

We performed batch effect removal on the GSE12452 dataset and the GSE53819 dataset and performed differential expression analysis on the matrix with batch effect removed, and obtained a total of 1646 differential genes (**Figures 2A, B**). The SUR1 and SUR2 modular genes and differential genes obtained from WGCNA analysis were screened using a Venn diagram to identify 410 overlapping genes (**Figure 2C**).

### lncRNA-mRNA co-expression network

**Figure 3:**
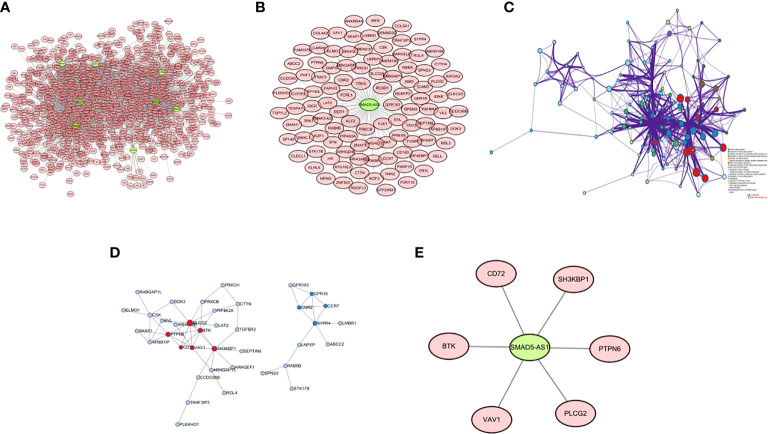
Construction of lncRNA-mRNA co-expression network and PPI network. **(A)** lncRNA-mRNA co-expression network of 8 lncRNAs in SUR1 and SUR2 modules; **(B)** key lncRNA SMAD5-AS1 and mRNA co-expression network; **(C)** PPI network of co-expressed mRNAs in the co-expression network; **(D)** MCODE plug-in screening the highest-scoring sub-network; **(E)** SMAD5-AS1 and hub mRNA co-expression networks.

There were 8 lncRNAs in the genes screened by the Wayne diagram, and the remaining 402 mRNAs were their target genes. The co-expression patterns of lncRNA-mRNAs in SUR1 and SUR2 modules were analyzed to construct co-expression networks, and 2203 co-expression relationships were obtained (**Figure 3A**). Among these 8 lncRNAs SMAD5-AS1 was significantly associated with the progression of NPC, and therefore SMAD5-AS1 was considered a key lncRNA. SMAD5-AS1 and 106 co-expressed mRNAs were got by degree screening (**Figure 3B**). The 106 mRNAs were analyzed using the Metascape tool to obtain PPI networks for further visualization of gene information and network construction (**Figure 3C**). The MCODE algorithm in Metascape clustered the PPI networks and screened the pivotal genes in the PPI networks (**Figure 3D**). Six mRNAs in the largest sub-network were regarded as key mRNAs. The co-expression network of SMAD5-AS1 and six hub mRNAs is displayed in (**Figure 3E**).

### Expression analysis

**Figure 4:**
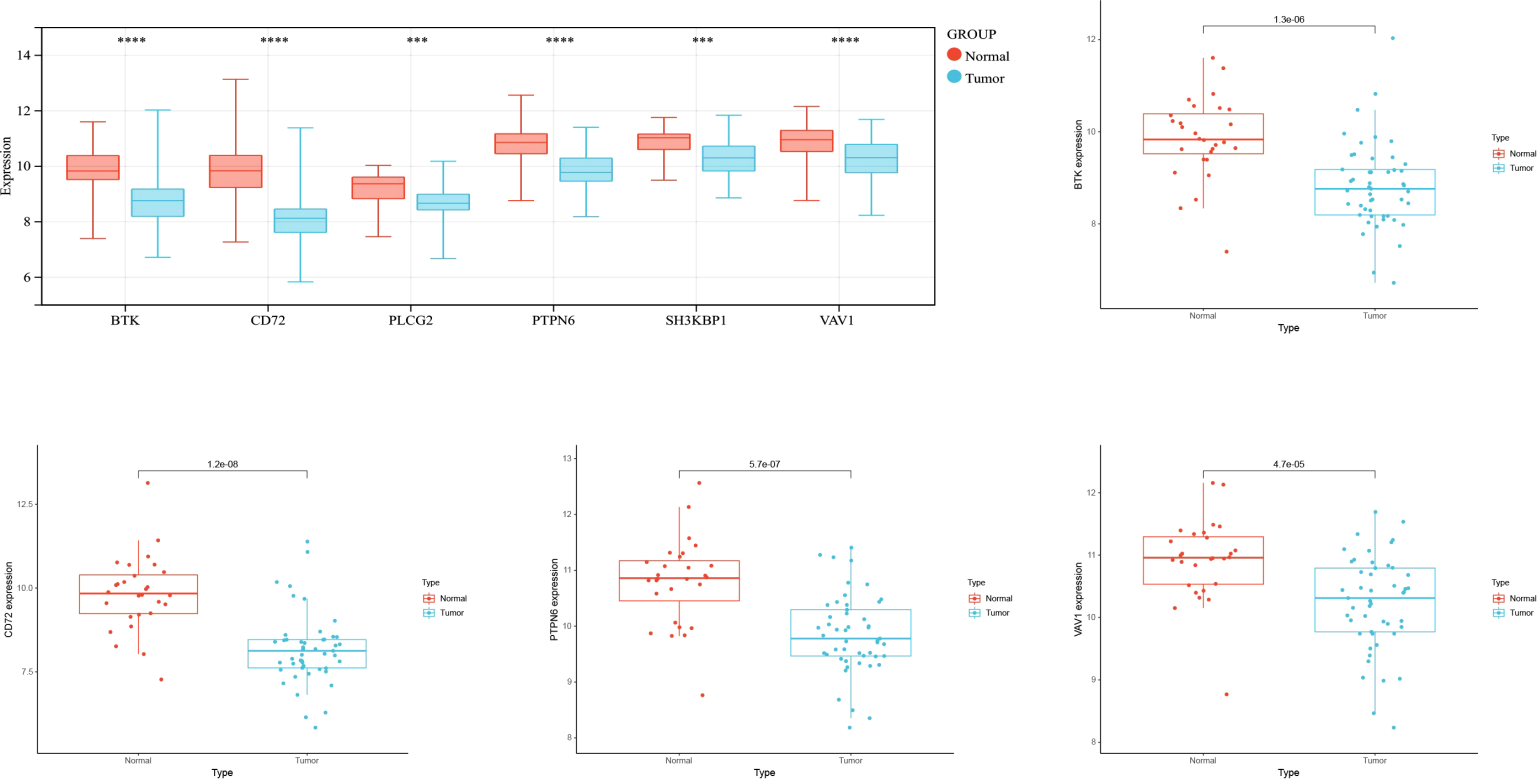
Expression analysis. ***indicates P < 0.001, ****indicates P < 0.0001.

To verify whether the screened hub genes were significantly related to NPC, the expression of genes in NPC tissues and normal tissues were analyzed, and the expression of BTK, CD72, PTPN6, and VAV1 was highly significant in cancer and normal tissues, and the expression in cancer tissues was lower than that in normal tissues (**Figure 4**).

### GSEA

**Figure 5:**
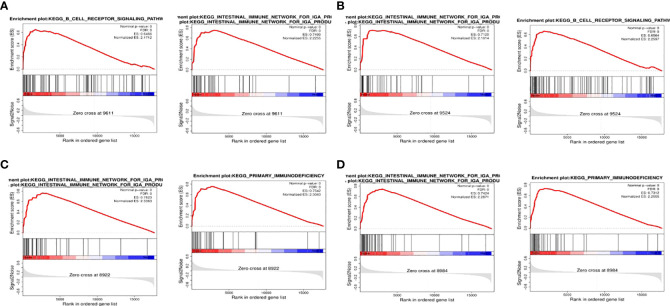
GSEA. **(A)** GSEA results of the top two correlations between BTK and signaling pathway; **(B)** GSEA results of the top two correlations between CD72 and signaling pathway; **(C)** GSEA results of the top two correlations between PTPN6 and signaling pathway; **(D)** GSEA results of the top two correlations between VAV1 and signaling pathway.

To detect the effect of genes on NPC, we classified the samples into two parts of high and low expression according to the expression of BTK, CD72, PTPN6, and VAV1, and analyzed the samples by GSEA. The top two most abundant signaling pathways or biological processes were listed according to the scores.GSEA confirmed that BTK and CD72 were mainly enriched in the IGA-producing intestinal immune network, B-cell receptor signaling pathway; PTPN6 and VAV1 were mainly associated with primary immunodeficiency, the IGA-producing intestinal immune network (**Figure 5**).

### qRT-PCR

**Figure 6:**
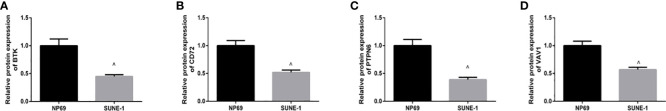
qRT-PCR. **(A)** Expression level of BTK; **(B)** expression level of CD72; **(C)** expression level of PTPN6; **(D)** expression level of VAV1. ^ indicates P<0.05 compared with the NP69 group.

We examined the expression of BTK, CD72, PTPN6, and VAV1 in NPC cell lines by qRT-PCR, and the expression levels of BTK, CD72, PTPN6, and VAV11 were lower in human nasopharyngeal carcinoma SUNE-1 cells compared with human nasopharyngeal epithelial cells NP69 (P<0.05) (**Figure 6**).

## Discussion

NPC is a common cancer of the head and neck (16). Currently, NPC is mostly squamous cell carcinoma, and the treatment of choice for NPC is radiotherapy (17, 18). Despite the continuous advances in radiotherapy, its distant metastasis remains the main reason for treatment failure, and the discovery of more efficient treatment methods is an essential future research direction. Thus, it is clear that understanding the etiology and mechanisms of NPC progression is important for the prevention and treatment of NPC.

In recent years, the direction of treatment for nasopharyngeal carcinoma has trended toward targeted therapy, less toxic and more effective forms of chemotherapy, and new technologies (19). lncRNAs, as regulators of biological functions, play key roles in various cellular and physiological processes (20) and are good choices in gene therapy. However, the lack of a comprehensive database providing resources for experimental validation of lncRNA function has become the most significant challenge in lncRNA-based therapeutic modalities.

In ceRNA theory, some lncRNAs can be co-expressed with the corresponding coding genes (15). We analyzed the lncRNA-mRNA co-expression network in NPC. We used the GSE12452 dataset and GSE53819 to build co-expression networks of clinical features and inter-module associations and selected the modules most relevant to NPC occurrence. Differential analysis was performed on both datasets after removing batch effects, and then overlapping genes between module genes and differential genes were screened out. Among the eight lncRNAs screened, SMAD5-AS1 was significantly associated with the biological progression of NPC cells (21), so it was considered a key lncRNA to construct a lncRNA-mRNA co-expression network related to NPC progression. Six hub mRNAs (BTK, CD72, PTPN6, VAV1, PLCG2, SH3KBP1) were then identified by the MCODE algorithm. The expression of genes in NPC tissues and normal tissues was observed, and it was found that the expression of BTK, CD72, PTPN6, and VAV1 was different in cancer and normal tissues, and the expression was lower in both cancer and normal tissues.

Bruton’s tyrosine kinase is a component of the B-cell receptor (BCR) signaling body (22). Activated BCR signaling contributes to the development of B-cell malignancies (23) and plays a role in the pathobiology of other hematologic malignancies such as chronic lymphocytic leukemia (CLL) (24). BTK, through the first-in-class inhibitor ibrutinib whose efficacy is clinically validated as a target for B-cell malignancies (25, 26). Our GSEA results also suggest that BTK is closely associated with the B-cell receptor signaling pathway. It was shown that BTK may be involved in the radioresistance process of NPC cells (27). This suggests to us that BTK may be a new key biomarker for NPC. CD72 is a co-receptor of BCR and an important regulator in the pathogenesis of several immune diseases; it plays a role in various B cell biological processes, including proliferation, apoptosis, and differentiation. In patients with Systemic Lupus Erythematosus, low expression of CD72 on B cells is negatively correlated with patient disease activity (SLEDAI) (28). CD72 is lowly expressed in multiple sclerosis and its ligand CD100 is increased in T cells (29). CD72 is lowly expressed in NPC tissues, suggesting that our low CD72 expression may be related to poor prognosis in NPC patients. Protein tyrosine phosphatase non-receptor type 6 is a key regulatory protein in cell signaling that regulates cell death and inflammation (30); It has different regulatory mechanisms and effects on cell cycle and cell proliferation in different tumors (31) and upregulated in colon cancer (32). We demonstrated low expression of PTPN6 in NPC by dataset analysis and qRT-PCR analysis, suggesting that our low expression of PTPN6 may be associated with poor prognosis in NPC patients. VAV1 is frequently mutated and overexpressed in hematopoietic malignancies and various cancers (33) and is accompanied by B-cell lymphoma. It was found that there is potential crosstalk between epithelial cells of VAV1 (which secrete CSF-1) and lymphocytes expressing CSF-1R, which leads to B-cell lymphoma (34). Through this mechanism, VAV1 promotes tumor propagation. No correlation between VAV1 and NPC progression has been found in existing studies, and our findings suggest that VAV1 is lowly expressed in NPC and that VAV1 is mainly associated with pathways of primary immunodeficiency, suggesting that VAV1 may be a new prognostic independent predictor for NPC patients.

Since lncRNAs can post-transcriptionally regulate target mRNAs, it is important to predict the interaction between the two and explore potential mechanisms of the pathological process through regulatory networks. Zhang J et al. characterized the regulatory networks of lncRNAs and mRNAs in cutaneous melanoma (SKCM) by analyzing expression profile data and identified potential therapeutic targets (35) A study by Ying-Juan Zheng found that SMAD5-AS1 knockdown inhibited EMT, cell proliferation, migration, and invasion in NPC by upregulating miR-106a-5p and downregulating SMAD5 (21). In our study, a novel NPC-regulated lncRNA SMAD5-AS1 mRNA axis was identified, which provides a reference for exploring the mechanism of NPC progression.

Taken together, our comprehensive analysis provides new way into the lncRNA-mRNA co-expression network in NPC progression. It suggests that lncRNA plays an important role in NPC and that lncRNA-SMAD5-AS1 may serve as a biomarker for predicting NPC prognosis. In addition, it may mediate the biological functions of NPC cells through co-expression networks involving BTK, CD72, PTPN6, and VAV1, which in turn affect the NPC process. The results obtained in this study may provide the necessary theory for future researches on the role of lncRNAs in NPC.

## Data availability statement

The original contributions presented in the study are included in the article/supplementary material. Further inquiries can be directed to the corresponding author.

## Author contributions

We contributed equally for this work.

## Funding

This work was supported by Ningbo First Hospital (2021KY981).

## Conflict of interest

The authors declare that the research was conducted in the absence of any commercial or financial relationships that could be construed as a potential conflict of interest.

## Publisher’s note

All claims expressed in this article are solely those of the authors and do not necessarily represent those of their affiliated organizations, or those of the publisher, the editors and the reviewers. Any product that may be evaluated in this article, or claim that may be made by its manufacturer, is not guaranteed or endorsed by the publisher.
